# A new principle of pulse detection based on terahertz wave plethysmography

**DOI:** 10.1038/s41598-022-09801-w

**Published:** 2022-04-15

**Authors:** Yu Rong, Panagiotis C. Theofanopoulos, Georgios C. Trichopoulos, Daniel W. Bliss

**Affiliations:** 1 Center for Wireless Information Systems and Computational Architectures (WISCA) at the School of Electrical, Computer and Energy Engineering, Arizona State University, Tempe, AZ 85281 USA; 2 Terahertz Research Lab at the School of Electrical, Computer and Energy Engineering, Arizona State University, Tempe, AZ 85281 USA

**Keywords:** Biomedical engineering, Electrical and electronic engineering, Optical techniques

## Abstract

This study presents findings in the terahertz (THz) frequency spectrum for non-contact cardiac sensing applications. Cardiac pulse information is simultaneously extracted using THz waves based on the established principles in electronics and optics. The first fundamental principle is micro-Doppler motion effect. This motion based method, primarily using coherent phase information from the radar receiver, has been widely exploited in microwave frequency bands and has recently found popularity in millimeter waves (mmWave) for breathe rate and heart rate detection. The second fundamental principle is reflectance based optical measurement using infrared or visible light. The variation in the light reflection is proportional to the volumetric change of the heart, often referred as photoplethysmography (PPG). Herein, we introduce the concept of terahertz-wave-plethysmography (TPG), which detects blood volume changes in the upper dermis tissue layer by measuring the reflectance of THz waves, similar to the existing remote PPG (rPPG) principle. The TPG principle is justified by scientific deduction, electromagnetic wave simulations and carefully designed experimental demonstrations. Additionally, pulse measurements from various peripheral body parts of interest (BOI), palm, inner elbow, temple, fingertip and forehead, are demonstrated using a wideband THz sensing system developed by the Terahertz Electronics Lab at Arizona State University, Tempe. Among the BOIs under test, it is found that the measurements from forehead BOI gives the best accuracy with mean heart rate (HR) estimation error 1.51 beats per minute (BPM) and standard deviation 1.08 BPM. The results validate the feasibility of TPG for direct pulse monitoring. A comparative study on pulse sensitivity is conducted between TPG and rPPG. The results indicate that the TPG contains more pulsatile information from the forehead BOI than that in the rPPG signals in regular office lighting condition and thus generate better heart rate estimation statistic in the form of empirical cumulative distribution function of HR estimation error. Last but not least, TPG penetrability test for covered skin is demonstrated using two types of garment materials commonly used in daily life.

## Introduction

Microwave (1–60 GHz) radars are widely used for detection of human vital signs, such as heart rate (HR), breathe rate (BR) and body temperature^1,2^, which are important biometrics for healthcare development^3–5^. Explicitly, these systems leverage advanced signal processing techniques such as complex signal demodulation^6^ and phase-based methods^7–12^ to extract vital signs from the captured backscattered signals. These methods were initially developed for narrowband systems and later extended to wideband radars offering better clutter performance. However, the robustness of these systems is hindered by certain limitations arising due to low operation frequency and limited radio frequency (RF) resources in these bands. The developed Doppler signal processing techniques have difficultly providing accurate pulse measurements when dynamic breathing pattern is present^13^, not to mention the presence of other sources of random body motion artifacts^14–16^. The fractional bandwidth (BW) of these frequencies leads to clutter noise due to low resolution range bins, especially in crowded environments/targets. For example, a 15% BW at 60 GHz is 9 GHz, leading to 1.7 centimeters (cm) range resolution. However, the heart motions on the body surface are less than 1 millimeter (mm), hence the signal of the cardiac pulses is hard to detect within such wide range bins that include breathing motions (a few mm to 1 cm^17^) and other body motions, along with clutter noise from other scatterers (e.g. clothing). In addition, the small apertures needed for mobile applications or embedded systems, lead to wide beams that capture the backscattered signals of multiple targets, further increasing clutter noise. Even though these issues are addressed exploring active motion cancellation and sensor fusion techniques^6,15,16,18,19^, still these configurations are limited in complex target scenes where multiple scatterers are located within in the field-of-view (FOV).

Alternatively, the use of higher-operating frequencies such as mmWave and THz (100 GHz–10 THz) could potentially alleviate the aforementioned limitations^20–25^. Namely, these non-ionizing frequencies offer large bandwidth that allow for increased range resolution, thus less cluttering noise. For instance, a 15% fractional BW at 300 GHz is 45 GHz, resulting into a 3.3 mm range resolution. In addition, small physical apertures are electrically larger in these frequencies (compared to microwaves) due to the small wavelength, leading to narrow beams that can be focused only on one target/person, thus further decreasing cluttering noise from undesired scatterers. Furthermore, the small motions caused by the pulse related surface skin motion and blood flow on/near the body surface, can be easily detected with phase-based methods, since the phase sensitivity increases due to the smaller wavelength. For example, a 0.5 mm motion causes a phase change of 3.6$^{\circ }$ at 60 GHz, while the respective phase change at 300 GHz is 18$^{\circ }$. This allows for the detection of heart caused micro-motions even in peripheral body sites based on mD motion effect^24^. Breathing-interference-free pulse measurement is possible at these sites because they are further away from the upper torso body area.

In combination with the aforementioned, these higher-operating frequencies are also exploited for high spatial resolution 3D imaging^26–29^ (both range and 2D cross-range), due to the directive beams and the large BW. Furthermore, instead of using complex and bulky mmWave/THz radars, low profile and complexity imagers can be utilized^30–32^ (e.g. reflecarrays) to form scannable narrow beams, offering more versatile vital signs monitoring (VSM) solutions.

In this work, a wideband sub-THz system is developed that forms narrow beams focusing the waves on different parts of the body and extract the pulse signals using the TPG concept based on the reflectivity changes in the magnitude response, rather than the mD phase^9,12,33^. As such, the recorded backscattered signals are leveraged to extract the vital signs of the person, using both the traditional micro-Doppler method and the herein introduced TPG. Located between the microwave and the optical frequency regions, THz waves constitute them a unique frequency band for remote vital sign sensing using different methods. Moreover, due to the nature of each technique (mD-motion based vs TPG-reflectance based detection), the micro motions and surface skin blood concentration of each body part contribute differently on each method’s accuracy. These measurement differences from the two methods are demonstrated and used as a cross-validation at the forehead BOI. Subsequently, the THz waves are focused on the temple, inner elbow, palm and fingertip to investigate their respective TPG vital sign detection capabilities. Distortionless vital sign sensing is possible by strategically choosing the body parts.

**Table 1:** Summary the key features of EM frequencies for vital sign detection.

VSM using EM waves				
Technology	Microwave^4–6,9–13,17,18,33,34^ (a few to a few tens of GHz)	THz^20–25^ (100–10000 GHz)	Near-Infrared/Infrared^35,36^	Visible Light^37–39^
Signal type	Phase, or complex	Magnitude, phase	Magnitude only	Magnitude only
Working principle	Motion based Doppler effect	Wave reflectivity$^{*}$/motion based Doppler effect	Mostly light reflectivity	Most light reflectivity
Algorithm performance	Low phase sensitivity	Good phase sensitivity	Good pulse sensitivity	Better pulse performance from more color channels
Vital signs detection	Breathing dominant/heartbeat not robust	Breathing and heartbeat separable	Heartbeat detectable/breathing not robust	Heartbeat detectable/breathing not robust
Computation load	Low	Low	High	High
Spatial resolution	Low (large aperture)	Good (millimeter resolution)	Excellent	Excellent
Clutter performance	Poor	Intermediate	Good	Good
Material penetration (e.g. clothes)	Excellent	Good^40,41^	Poor	Non-existent
Sensing in low visibility conditions (e.g. smoke, fog)	Excellent	Excellent	Good	Poor
Synergy with radar imaging (e.g. NLOS)	Poor (coarse imaging)	Excellent	None	None
Cost and manufacturing effort	Cheap	Very high	High	Very cheap
Privacy issue	None	None	Mild	Yes

### VSM in EM waves

The EM spectra for VSM can be broadly divided into four categories, microwave (including mmWave), THz, infrared and visible light. Due to the aforementioned reasons, there is significantly less effort on VSM using THz. Before presenting the new insights on using THz for VSM, it is worthwhile to review the current advances of mD bio-radar sensors and rPPG optical sensors.

rPPG using optical sensors (infrared and visible light) are advantageous over microwave bio-radars for motion tolerance VSM. That is because rPPG signal is based on the changes in the skin reflectivity (or skin color shift) not from skin motion. High resolution images from optical sensors provide abundant information for signal processing. Other body motion artifacts can be separated via computer vision techniques by leveraging millions of image pixels, multiple available color channels from low-cost webcams. Infrared is heavily investigated due to privacy issue of using normal color cameras. In general, optical sensors do not penetrate many common materials, including clothes and blankets and are limited to line-of-sight (LOS) applications.

On the other hand, conventional narrowband mD radars with limited array size (mostly single antenna systems) are not able to handle realistic dynamic motion profile. Popular Doppler phase signal is more susceptible to chest motion and other random body movements, which are significantly stronger than the pulse signal in the radar return due to larger radar-cross-section and physical displacement. Direct pulse measurement from chest area is not possible without suppressing respiration motion and naive spectral separation is not sufficient for HR estimation due to the breathing and heartbeat coupling effect^13^. Active motion cancellation techniques considers ultra-wideband (UWB) ^14,42^, dual-radars^6,16^ and RF front end re-design^18^ producing encouraging results but their effectiveness needs further investigations.

Recently a THz system^24^ is shown for pulse detection at peripheral body sites because of excellent phase sensitivity due to smaller wavelength. Breathing-free pulse measurement is achievable at major peripheral artery sites, such as wrist, with large BW and focusing beam at THz. Furthermore, this study goes one step further and demonstrates in the following sections measurable plethysmographic signals from the face and other body parts in the THz magnitude response. Therefore, this new observation is named Terahertz-Wave-Plethysmography (TPG). A high-level comparison of VSM using EM waves is tabulated in Table 1.

## Terahertz-wave-plethysmography (TPG)

**Figure 1:**
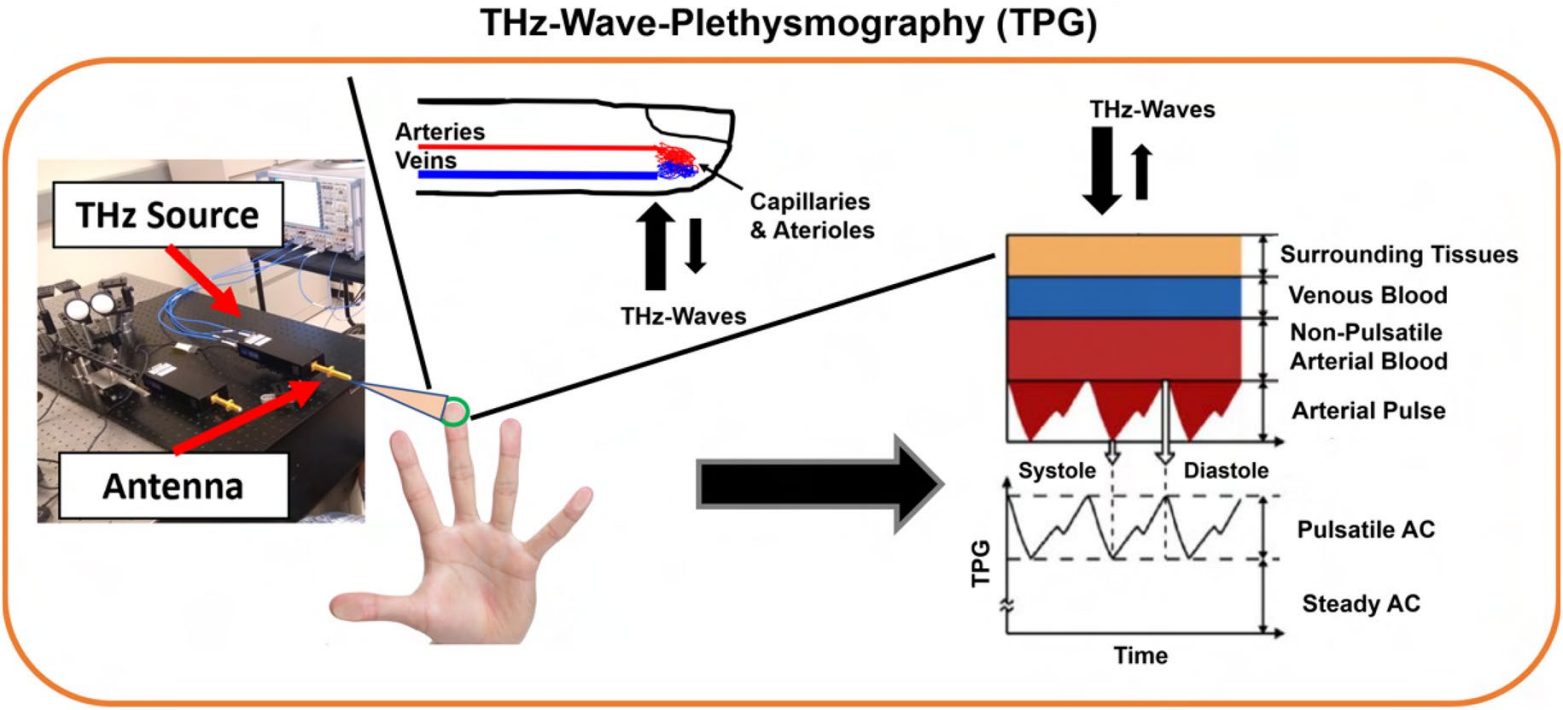
Working principle of THz-wave-plethysmography (TPG).

A novel concept of TPG is described in Fig. 1. TPG detects blood volume changes in the dermis layer by measuring the reflectance of THz wave, similar to the PPG principle. According to references^27,43,44^, THz wave can reach the dermis layer through out the peripheral body parts. Similar skin optical properties found in NIR and visible light for plethysmography also found in THz waves^45,46^, such that THz interacts with hemoglobin in blood cell. There are measurable differences in the spectra of blood and its components when the hemoglobin content changes in the THz frequencies. It therefore can be inferred that the pulsatile variation exists in the THz wave absorption in an illuminated skin area caused by the difference in absorption curves of oxygenated and deoxygenated blood, and thus TPG is possible. In the following, EM simulation and human subject experimental results are presented to validate the proposed theory.

The wideband THz sensing setup includes a vector network analyzer (VNA, model: Rohde Schwarz ZVA24) and a frequency extension module (model: Virginia Diodes WR3.4) that up-converts the microwave signals from the VNA to the THz band (220–330 GHz, in which 30 GHz bandwidth is used from 285 to 315 GHz). A diagonal horn antenna with a gain of 26 dB is integrated to the extender to emit THz signals to the free space such that the waves are formed into a narrow beam with a half-power beam width of approximately 10$^{\circ }$. As such, the beam illuminates the region of interest (ROI) of human body and the backscattered signals are recorded by measuring the $\textit{S}_{11}$ parameter.

### EM simulation study

THz measurements are compared with theoretical data validating the use of reflectivity for the extraction of pulse. The reflectivity-based process is commonly used in the optical spectrum, where an infrared emitter or ambient light illuminates the skin and the intensity of the backscattered lights is modulated^39,47,48^. Using the time-variant magnitude response of the reflected signals, the HR can be extracted.

**Figure 2:**
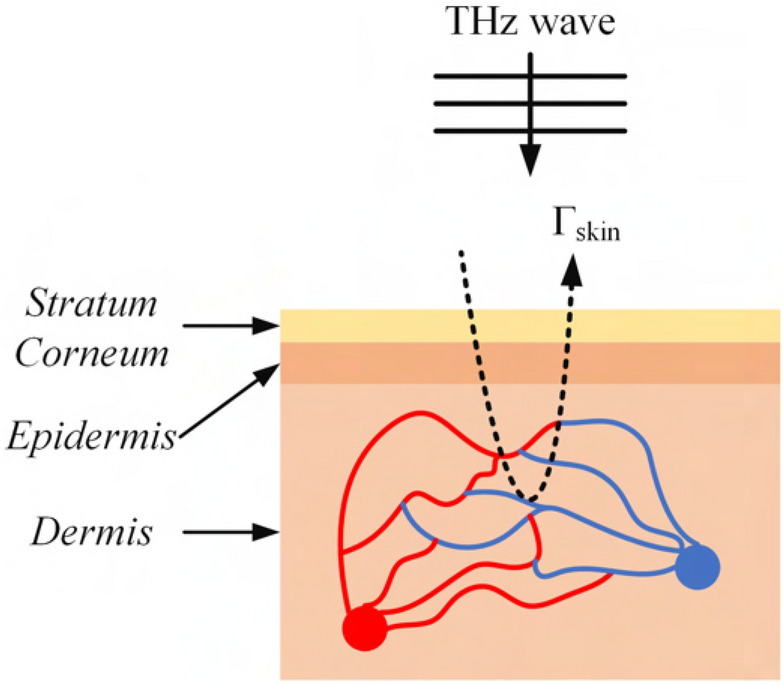
The upper skin structure during the cardiac cycle. In the systole phase, the blood volume is smaller leading to lower tissue conductivity. On the contrary, during the diastole, the arteries and capillaries expand, leading to more blood volume within the tissue, thus higher conductivity.

According to references^39,48^, this modulation in reflectivity has a twofold cause. Firstly, the amount of blood present in the subcutaneous skin vessels and capillaries changes leading to more blood (thus more losses) in the reflected waves. The second cause of this modulation is blood consistency. Namely, the amount of oxygen in the blood varies within the cardiac cycle and the losses of the EM waves are proportional to this variation. For example, the sensitivity of green light radiation to the oxygen levels in the blood is well established enabling the use of green light sensors for the detection of pulse^39,47,48^. However, in a recent study, it was demonstrated using THz spectroscopy that waves ranging from 0.1 to 1 THz are also sensitive to the consistency of blood^46^. Thus, in this case study, it is assumed that the magnitude modulation in the measurements is attributed to the sub-skin conductivity variation, caused both by the amount of blood in the subcutaneous capillaries and the water concentration in it.

To study the reflectivity modulation caused by the cardiovascular activity at the peripheral body sites, the skin model shown in Fig. 2 is considered. The skin is modeled as a three-layered structure^41,49^: the top part is the thin layer of the stratum corneum, followed by the epidermis where the presence of capillaries is very limited, and finally the dermis which is modeled as a semi-infinite layer. The EM material properties of these layers are tabulated in Table 2. As such, the stratum corneum and the epidermis have low conductivity and the dermis is a conductive layer since it is filled with capillaries. During the cardiac cycle, the variation of blood volume is assumed to modulate the conductivity of the dermis, leading to the modulation of the reflected THz waves.

**Table 2:** Skin model parameters.

	Thickness	$\eta _{r}$	$\sigma _{diastole}$ (S/m)	$\sigma _{systole}$ (S/m)
Stratum corneum (sc)	5	2.4	$10^{-5}$	$10^{-5}$
Epidermis (ep)	90	3.2	1	1
Dermis (derm)	Indefinite-half-space	3.9	41	36

The dermis layer conductivity as given in Table 2, is calculated by,

1 $$\begin{aligned} \sigma _{dermis} = (1 - \xi ) \, \sigma _{skin,dry} + \varepsilon \, \sigma _{blood}, \end{aligned}$$

where $\sigma _{skin,dry}$ is the dry skin conductivity^50^, $\sigma _{blood}$ is the blood conductivity^50^, and $\xi$ is the blood concentration. For this case study, the blood concentration is 60% during the systole and 70% during the diastole^39^. Then, the reflection coefficient of the skin is given by^51^,

2 $$\begin{aligned} \Gamma _{skin} = \frac{r_{1} + r_{2}z_{1} + r_{1}r_{2}r_{3}z_{2} + r_{3}z_{1}z_{2}}{1 + r_{1}r_{2}z_{1} + r_{2}r_{3}z_{2} + r_{1}r_{3}z_{1}z_{2}}, \end{aligned}$$

where

3 $$\begin{aligned} r_{1}&= \frac{\eta _{sc}-\eta _{air}}{\eta _{sc}+\eta _{air}} \end{aligned}$$

4 $$\begin{aligned} r_{2}&= \frac{\eta _{ep}-\eta _{sc}}{\eta _{ep}+\eta _{sc}} \end{aligned}$$

5 $$\begin{aligned} r_{3}&= \frac{\eta _{derm}-\eta _{ep}}{\eta _{derm}+\eta _{sc}} , \end{aligned}$$

are the partial reflection coefficients at the air and the stratum corneum interface, the stratum corneum and the the epidermis interface, and the the epidermis interface and the dermis interface. And,

6 $$\begin{aligned} z_{1}&= e^{-2ik_{sc}t_{sc}} \end{aligned}$$

7 $$\begin{aligned} z_{2}&= e^{-2ik_{ep}t_{ep}} \end{aligned}$$

8 $$\begin{aligned} z_{3}&= e^{-2ik_{derm}t_{derm}} , \end{aligned}$$

are the propagation delay in stratum corneum, epidermis and dermis respectively, where

9 $$\begin{aligned} \eta _{air}&= \sqrt{\varepsilon _{0}} \end{aligned}$$

10 $$\begin{aligned} \eta _{x}&= \sqrt{\varepsilon _{x}(1 - i \frac{\sigma _{x}}{2\pi f \varepsilon _{x}})}, \end{aligned}$$

and

11 $$\begin{aligned} k_{x} = \frac{2\pi f \sqrt{\varepsilon _{x}}}{c}, \,\,\,\,\, x = sc, ep\text {, or }derm. \end{aligned}$$

where the subscript *x* denotes either the stratum corneum, the epidermis, or the dermis, *f* the frequency, $\varepsilon _{0}$ the free space permittivity, and *c* the speed of light in free space.

The aforementioned equations define the reflection coefficient under plane wave illumination for a specific value of $\xi$. However, the value of the $\xi$ can vary between 60 and 70% during the cardiac cycle^39^. Thus, the $\xi$ of (2) is defined as

12 $$\begin{aligned} \xi = 0.65+0.05 \, \mathrm{{cos}} (2 \,\pi \,\alpha \,t\,/ 60 ), \end{aligned}$$

where *t* is the time in seconds and $\alpha$ denotes HR. As such, for every time instance within the cardiac cycle, a different $\xi$ value is calculated and the respective reflection coefficient ($\Gamma _{skin}$) is computed using (2)–(11). The reflection coefficient can be calculated for various values of *t* and *f*, creating a 2D vector $\Gamma _{skin}(f,t)$. This complex set of values are then used to carry out the computation of the HR using the TPG algorithm, by using the magnitude of the complex reflection coefficient for every (*f*, *t*) pair.

**Figure 3:**
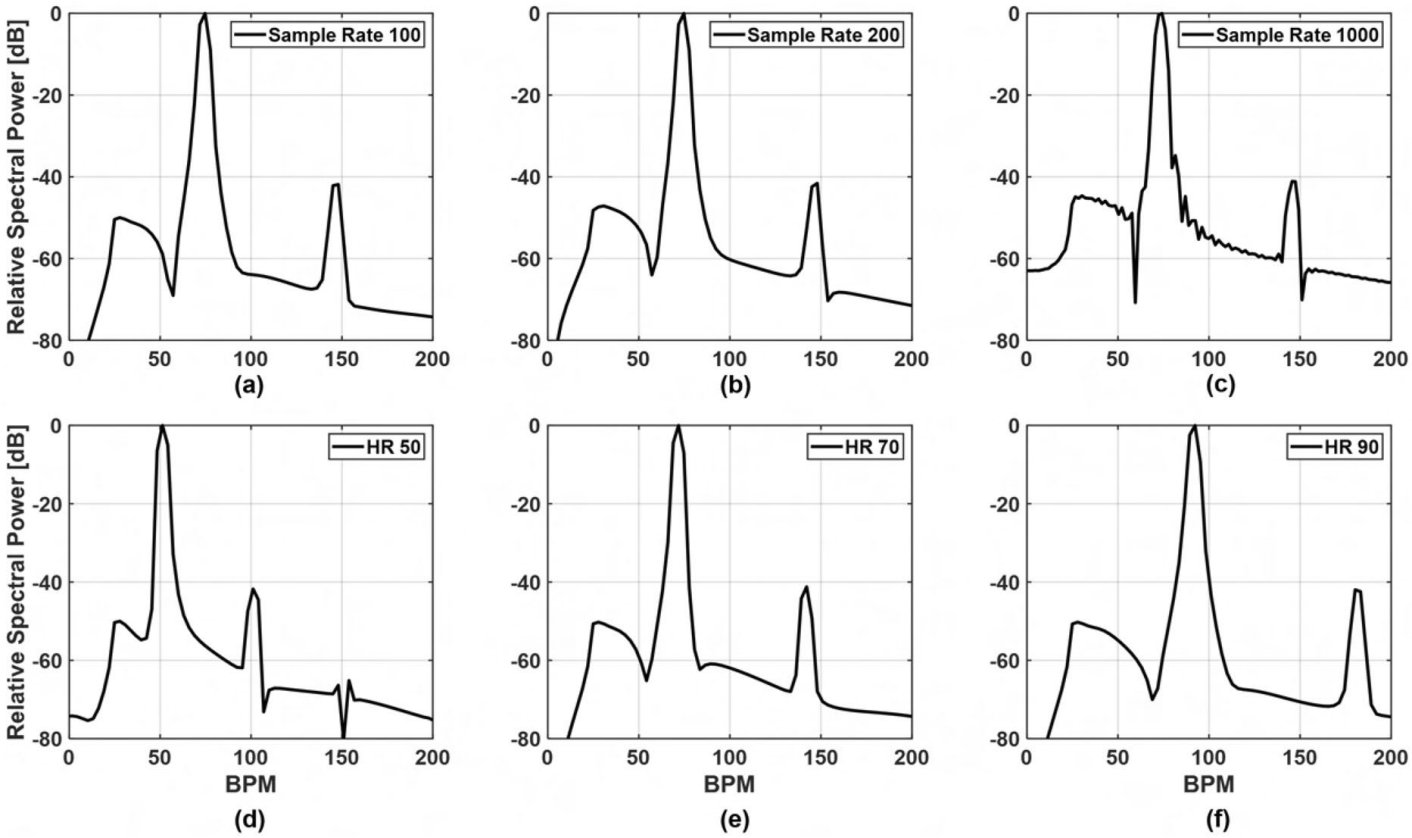
Spectrum from simulated data as function of sample rate and blood flow cycles. The first row (**a**–**c**) shows the spectra from sample rate (samples/s) at 100, 200 and 1000 with blood flow cycles fixed at 72 cycles per minute; the second row (**d**–**f**) the spectra at 50 cycles, 70 cycles, 90 cycles with a fix sample rate of 100.

**Figure 4:**
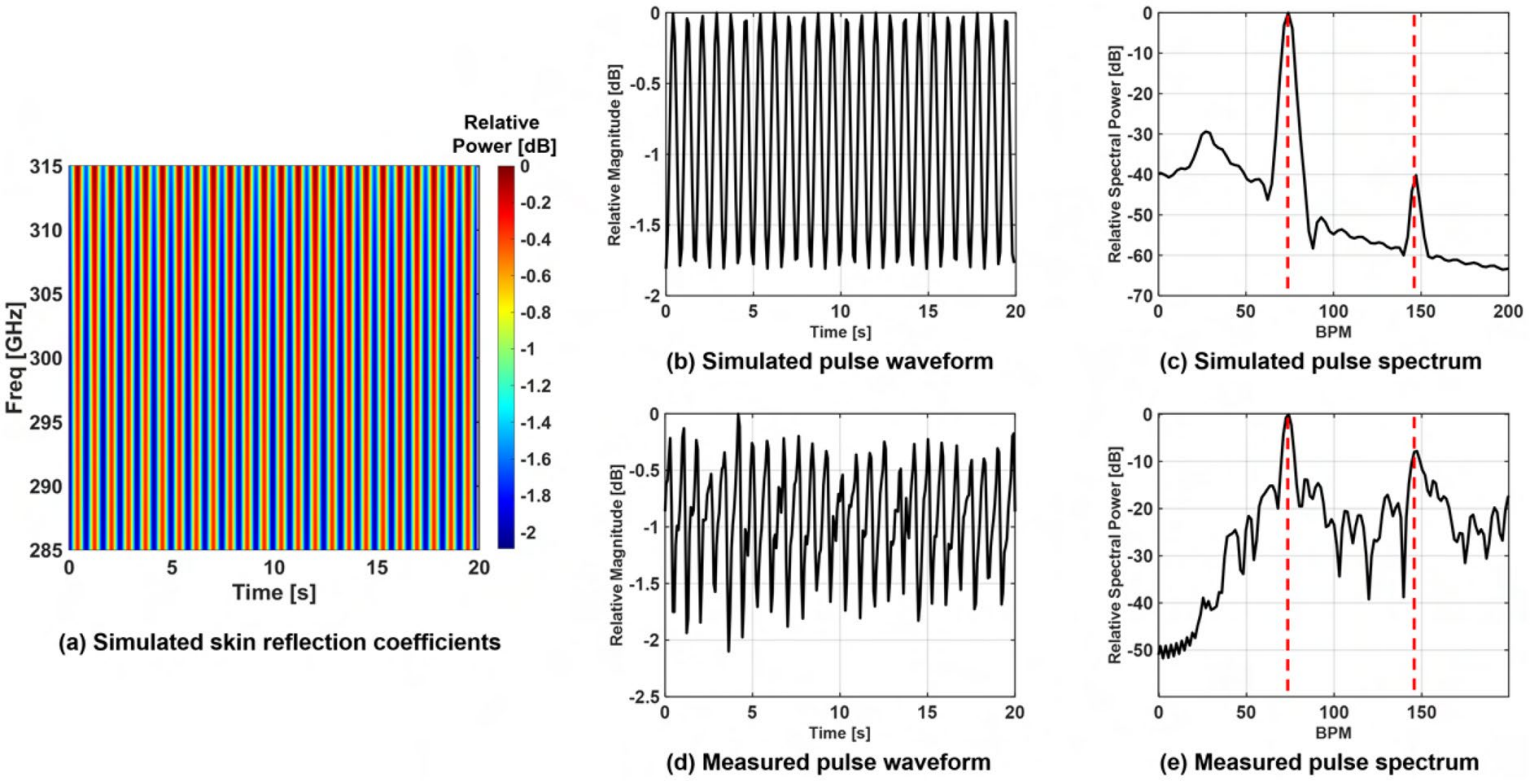
(**a**) The simulated skin reflection coefficients from time-varying dermis conductivity values due to cyclical blood flow (computer simulated at 72 cycles per minute) in the 285–315 GHz spectrum; (**b**) the corresponding pulse waveform extracted by averaging the simulated skin reflection coefficients across the frequency range; (**c**) the corresponding pulse frequency spectrum of the simulated data; (**d**) the reference THz pulse measurement in 285–315 GHz spectrum taken from a subject’s forehead; (**d**) the corresponding pulse frequency spectrum of the measured data. The peak-to-valley variation of the measurements (**d**) is similar to the one observed by the simulated data (**b**) approximately 1.8 dB and also the fundamental and second-order heartbeat harmonics locations are matched in the simulation data (**c**) and the measurement data (**e**), thus validating the working principle of the novel TPG measurement approach.

TPG frequency spectra are displayed in Fig. 3a–c. The skin’s reflection coefficient is calculated in the 285–315 GHz range with a step of 214.3 MHz and a HR of 72 BPM, for various time sampling rates. As shown, the sampling rate does not affect the two peaks produced by the TPG algorithm, which correspond to the fundamental and 2nd-order harmonic of the HR, respectively. The frequency separation between the first and the second peak in the TPG spectra varies as the HR changes in Fig. 3d–f, thus validating that the second peak in the TPG spectra is a harmonic of the HR, produced by the non-linearity of (6). The proposed mathematical model, is further validated by the results presented in Fig. 4. The magnitude of the calculated $\Gamma _{skin}$ in the 285–315 GHz range within a 20 s window and a HR of 72 BPM is depicted in Fig. 4a. Sampling time and duration of the numerical experiment are the same as the one used in the measurement campaign. The simulated pulse waveforms and spectra are shown in Fig. 4b,c, and the corresponding measurements in Fig. 4d,e. As such, the peak-to-valley modulation on the THz reflection coefficient during the cardiac cycle is close to 1.8 dB in Fig. 4b, which is in agreement with the THz measurements presented in Fig. 4d. The THz measurement in Fig. 4d is post-processed by applying a bandpass filter to remove the static DC component and lower frequency component. The agreement is also found spectrally by inspecting the pulse frequency energy concentration in Fig. 4c,e. Therefore, above theoretical approach verifies that the blood concentration in the upper layers of the skin, during the cardiac cycle, leads to the measurable reflectivity change in the THz frequency range. This effect enables pulse detection using the THz reflectivity measurements, which are not dominated by the breathing motions and other body motions, thus, leading to a robust pulse monitoring tool.

**Figure 5:**
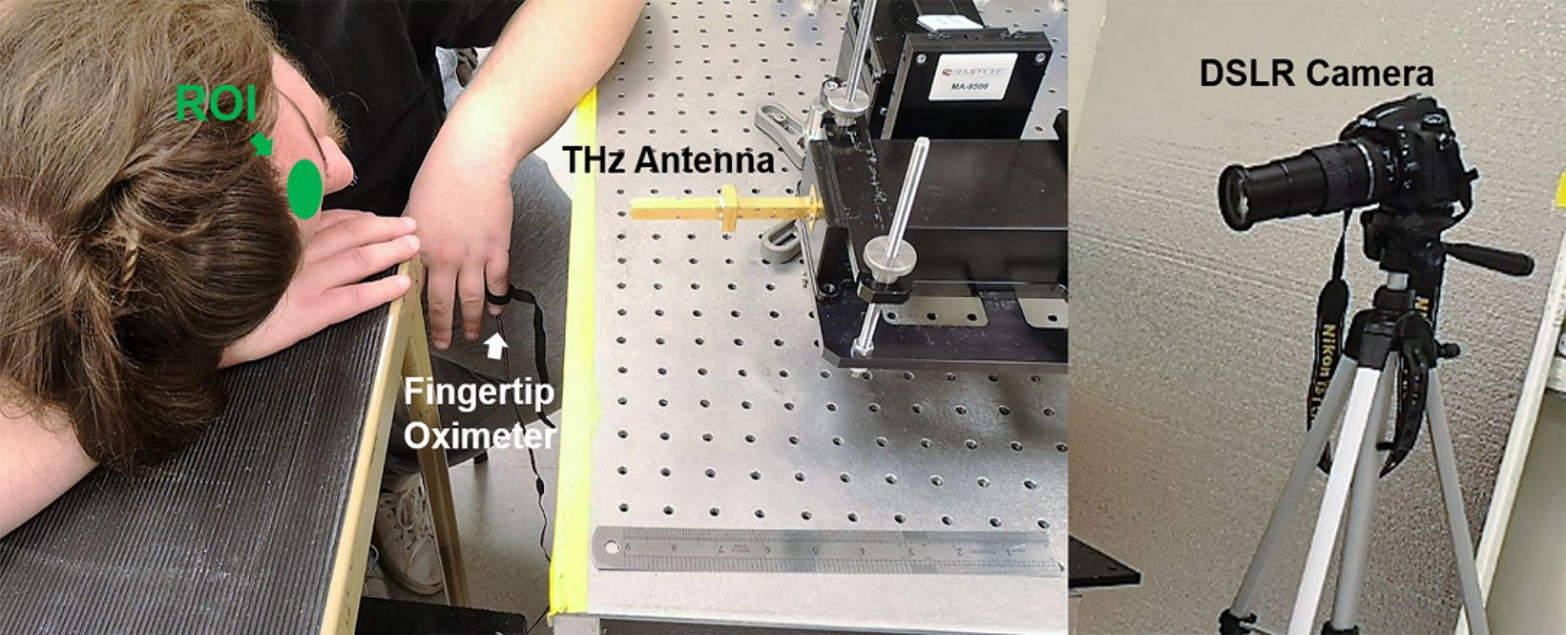
Measurement setup at forehead region of interest (ROI) as indicated in the green area. A fingertip oximeter is used for providing contact PPG signal. Simultaneously, a digital single-lens reflex (DSLR) camera Nikon D750 is focused at the forehead area and recording at 30 frame per second with 1920 $\times$ 1080 pixel resolution. The rPPG signal is extracted by processing the sequence of images.

**Figure 6:**
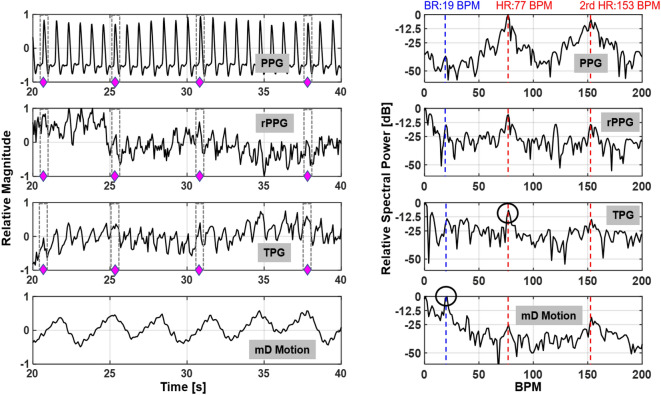
Waveform comparison from different measurement sensor outputs and their associated spectra. The left column shows temporal waveform of PPG, rPPG, TPG and mD phase; the right column their spectra at the frequency of interest. The measurement are synchronized as indicated by the diamond markers. Major spectral components related to pulse and breathing are labeled by blue and red vertical lines. The higher-order harmonic of heartbeat is highlighted for cross-validation of the fundamental heartbeat spectral location.

**Figure 7:**
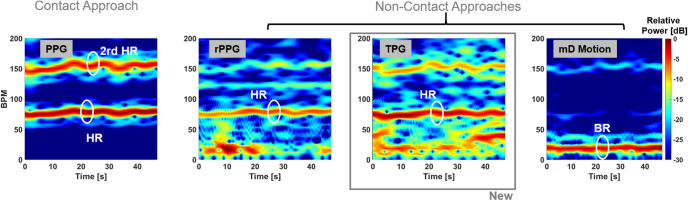
Time-frequency representation of the different measurement data. From the left to the right: spectrograms of PPG, rPPG, TPG, and mD phase. Based on the operation mode, they are divided into two categories: contact approach, PPG, and non-contact approaches including rPPG, TPG and mD phase. PPG, rPPG and TPG show similar spectral structures, in which the fundamental heartbeat and second-order harmonic of heartbeat are clearly visible and highlighted. The fundamental heartbeat in rPPG and TPG is the most significantly spectral energy. Motion artifacts show up in rPPG and TPG in the form of lower frequency interference close to DC due to random body movement and breathing but they are not dominant. By inspecting the TPG and the mD motion results, it validates the underline principle that the novel TPG measurement is mostly a magnitude-modulated reflectance measurement. PPG is a contact technique and requires direct skin contact. In this example, the measurement distances for the remote techniques: rPPG, TPG and mD motion are 2 m, 30 cm and 30 cm, respectively.

### TPG measurements

TPG describes THz wave reflectivity measurement and is obtained from the magnitude response of received signal in time domain. TPG and mD motion, measurement from phase, are simultaneously extracted using the proposed wideband THz system. Representative TPG and mD motion measurements along with multiple types of reference measurements are displayed in Fig. 6. In this example, the forehead of the test subject is illuminated by the THz sensing system. It can be concluded that the magnitude variation mostly corresponds to the THz reflectivity change, similar to the PPG principle. On the other hand, the extracted phase variation of the THz return is breathing-motion dominant and it captures the skin surface vibration, inner tissue movement at the top dermis layer due to pulsation and slightly head movement due to breathing activity. Physiological motion is a quasi-periodic narrowband signal. The fundamental frequency signatures such HR and BR change slowly over time. Therefore, in a short processing window, HR and BR can be estimated by inspecting the peak spectral energy location at the proper frequency regions.

To avoid any man-made artifact and maintain purity of the significant signal components, only mean centered, normalized magnitude and phase signals without additional filtering are used to demonstrate the advantages of TPG measurements. The experimental setup is illustrated in Fig. 5, in which the reference signals PPG and rPPG are acquired simultaneously. THz measurements and their spectra are compared in Fig. 6. The left column represents, from top to bottom, the PPG waveform, the rPPG waveform, the TPG waveform and finally the mD phase waveform. Minimum processing is applied on each sensor output (scaling) for visualization. The four different types of measurements are aligned. The pulsation signal is the dominant trend in PPG, rPPG and TPG, because they are all reflectivity based measurements. The diamond markers indicate aligned individual pulses in PPG, rPPG, and TPG. While the mD phase is motion sensitive and is breathing-motion dominant. This observation is consistent with microwave and mmWave radars for VSM^13,33^. The corresponding vital sign spectra are shown in the right column of Fig. 6. No filtering is applied for generating the spectra and only a hanning window is used before taking Fourier transform to suppress sidelobes. Similarly, the major spectral components in PPG, rPPG and TPG are fundamental HR and the 2nd-order harmonics of HR. Except strong DC component, the dominant spectral energy in mD phase is BR. The blue vertical and red vertical lines represent the reference BR and HR and its harmonics. In Fig. 6 second column last row, the highlighted breathing component is about 25 dB stronger than the possible fundamental pulse component and thus makes it challenging for robust pulse measurement, which is still an open question in microwave radar VSM.

Additionally, the time-frequency analysis is applied to the same dataset. The spectrograms in Fig. 7 are generated using short-time Fourier transform with a sliding window 13-s and one sample increment. The y-axis is the frequency in beats per minute (BPM). An infinite impulse response high pass filter with cut-off frequency 0.1 Hz is applied in the y-axis direction to remove the zero frequency component (DC) to emphasize the spectral energies of interest. Overall, the contact approach PPG gives best performance, which is used as the standard pulse reference but it requires direct physical contact neutralizing the motivation of remote sensing. On the other hand, the results from the three different non-contract methods provide distinct implications. TPG is similar to the rPPG as the pulse signal almost maintains the stronger spectral components during the experiment. Compared to PPG, rPPG and TPG experience some low frequency interference due to involuntary body motion and breathing motion. These motion artifacts are not constant and dominant thus can be easily separated through post-processing. The mD phase measurement is known for motion sensitivity and captures breathing motion consistently during the experiment.

Several important observations can be made based on this carefully designed experiments. The breathing signal is weakly present at the TPG measurement and it validates that the magnitude change originates mostly from the variations in skin conductivity. Plethysmography using THz wave, therefore, is feasible and our study helps demystify the origin of non-contact reflectance plethysmography. So far the community have not reached consensus on the physical principles of rPPG^52^. At least two hypotheses on the causes of the observed phenomenon are: (1) optical density change within the tissue caused by arterial pulsations and (2) local deformation of tissue caused by capillaries^39^. Or put it another way, one is EM wave reflectivity change and the other one is local micro-tissue motion. Note that the local micro-tissue motion is a much smaller physical displacement compared the body motion related to respiratory activity, which in this study shows up in the phase-based (motion sensitive) method. Absence of a stronger breathing component in the TPG waveform and spectrum concludes that the local tissue motion is not the leading cause of the detected pulse in the magnitude response since the large-scale breathing motion is much stronger than the micro-scale local tissue motion.

### Accuracy

**Figure 8:**
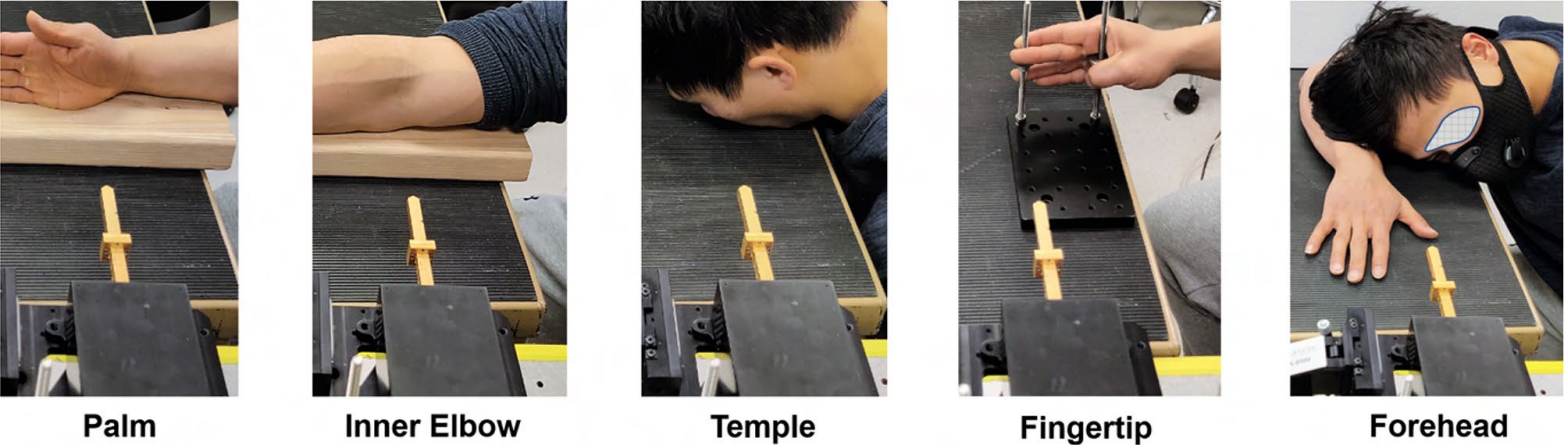
TPG measurement setups at various peripheral body ROIs, including palm, inner elbow, temple, fingertip and forehead. (Informed consent is obtained from this test subject for publication of identifying image.).

**Figure 9:**
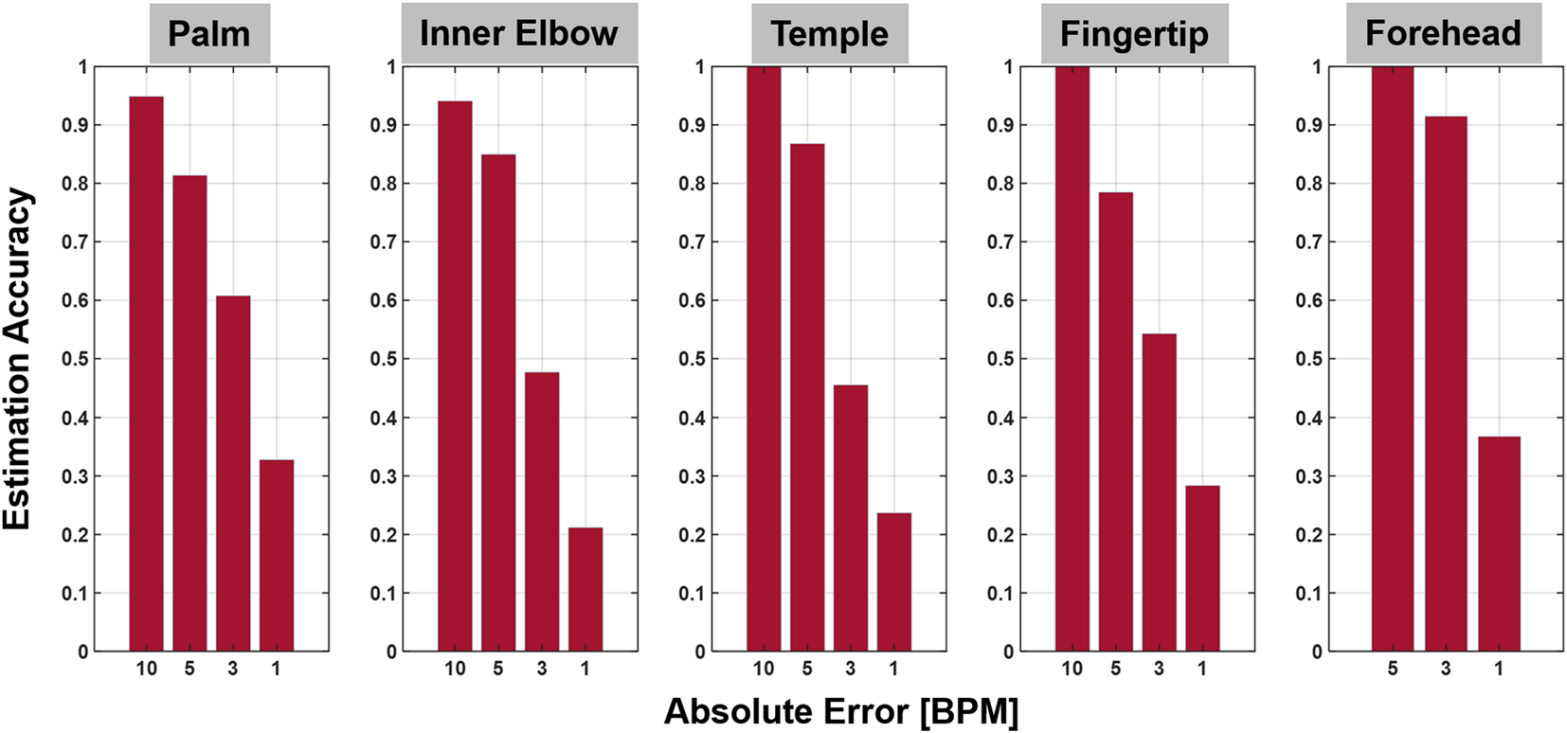
HR estimation error histograms at various peripheral body ROIs in Fig. 8. Each histogram shows the percentage of HR estimates that its estimation error is within ($\le$) 10, 5, 3 and 1 BPM respectively. Palm: 94.9$\%$ $\le$ 10, 81.4$\%$ $\le$ 5, 60.8$\%$ $\le$ 3, 32.8$\%$ $\le$ 1; inner elbow: 94.1$\%$, 85.0$\%$, 47.7$\%$, 21.2$\%$; temple: 100$\%$, 86.8$\%$, 45.6$\%$, 23.7$\%$; fingertip: 100$\%$, 78.5$\%$, 54.3$\%$, 28.4$\%$; forehead: 100$\%$, 91.5$\%$, 36.8$\%$.

**Table 3:** HR estimation performance at peripheral body sites.

	Palm	Inner elbow	Temple	Fingertip	Forehead
Mean (BPM)	2.62	3.53	2.97	2.75	1.51
STD (BPM)	3.35	3.33	3.41	2.39	1.08

The accuracy of TPG measurements is demonstrated at five exemplary body sites: palm, inner elbow, temple, fingertip and forehead. These experiments were performed at ASU Terahertz Research Lab. The setups and targeted ROIs are illustrated in Fig. 8. During the experiment, the test subjects were instructed to breath normally and maintain stationary in a relaxing state. However, random body motion and involuntary movements were observed during data acquisition and in reality they are inevitable especially when the experiment time increases.

Four 240-s datasets are used for HR error analysis. The results in Fig. 9 are generated with a sliding window of 13-s with one sample increment. The HR estimation error histogram displays the error distribution at four different levels. The HR estimation performance is calculated as the percentage of HR estimates that the corresponding estimation error is within ($\le$) 10, 5, 3 and 1 BPM, respectively. At palm, the measurement error distribution is 94.9$\%$ $\le$ 10, 81.4$\%$ $\le$ 5, 60.8$\%$ $\le$ 3, 32.8$\%$ $\le$ 1; inner elbow: 94.1$\%$ $\le$ 10, 85.0$\%$ $\le$ 5, 47.7$\%$ $\le$ 3, 21.2$\%$ $\le$ 1; temple: 100$\%$ $\le$ 10, 86.8$\%$ $\le$ 5, 45.6$\%$ $\le$ 3, 23.7$\%$ $\le$ 1; fingertip: 100$\%$ $\le$ 10, 78.5$\%$ $\le$ 5, 54.3$\%$ $\le$ 3, 28.4$\%$ $\le$ 1; forehead: 100$\%$ $\le$ 5, 91.5$\%$ $\le$ 3, 36.8$\%$ $\le$ 1. Overall, on average the error statistics: at palm, inner elbow, temple, fingertip and forehead are summarized in Table 3. HR estimation accuracy from forehead has a mean error 1.51 BPM and STD 1.08 BPM, and is superior to the other four BOIs because the larger surface area at forehead and better upper body stabilization in prone position as shown in Fig. 5. These results together validate the feasibility of TPG principle for direct pulse monitoring.

## Discussion

In this study, the feasibility of radar plethysmography was investigated using THz waves. Electrocardiac activities measurement device ECG and contact PPG measurement device are gold standards to measure pulse/HR. The emerging remote sensing technologies using radar and vision sensors transfer the way of measuring the diversity of physiological implications of human body. We have presented a comprehensive review of operating principles and experimental results of the two exciting technologies. For non-disturbance, ubiquitousness, all-weather, penetrability, privacy-preserving sensing requirements, the radar technology is favored in these perspectives. We proposed a novel concept of TPG to extract pulse information analogous to the known optical principle PPG.

THz radar system detect cardiac pulse based on plethysmography principle in addition to the mD principle was investigated by a multiplicity of measurements. Exemplary validation measurements show high similarities between radar TPG signal and reference contact-PPG signal regarding the R-peak locations and the spectral peak location. The presented comparison between radar TPG, and mD motion, rPPG, PPG proves the feasibility of radar-based plethysmography detection. Our analysis considered the differences regarding measurement principles, sizes of the measurement spots, and BOIs. Increased HR estimation error is observed at some BOIs. It is caused by the lower signal-to-noise-ratio (SNR) of the TPG, which can be explained by the surface curvature and area of the measurement spots and measurement stabilization, and which is substantiated by the higher variations of HR estimates. The TPG HR estimation performance can be enhanced by system-level optimization, such as improvement of the dynamic range and emission power of the utilized radar system, and processing optimization. These considerations are not the focus of this study and will be investigated in separate efforts.

Further more, the new insight of cardiac physiology of THz waves interaction with human body at various BOIs improves the direct pulse monitoring performance in a non-contact fashion. The conventional mD approach focuses at the chest area. It generates noisy and inaccurate signal highly distorted by stronger body movement and breathing motion. Direct pulse monitoring and instantaneous inspection are not feasible using conventional approaches. Recently, research and technology in the field of THz science and electronics has undergone tremendous development, for example THz human body imager^26–29^. Being able to use high spatial resolution THz images to strategically detect pulse information, through clothing or bedding, from multiple spots of human body opens new opportunities for biomedical applications using THz waves: inspecting blood circulation, extracting blood pressure related biometrics such as blood pulse pressure, pulse wave velocity. The unique features of THz waves, such that they exhibit electron-like and photon-like properties, implies two different ways of VSM. For the first time, radar technology is proven to be able to detect pulse signal using optical principle.

## Methods

### Radar processing

**Figure 10:**
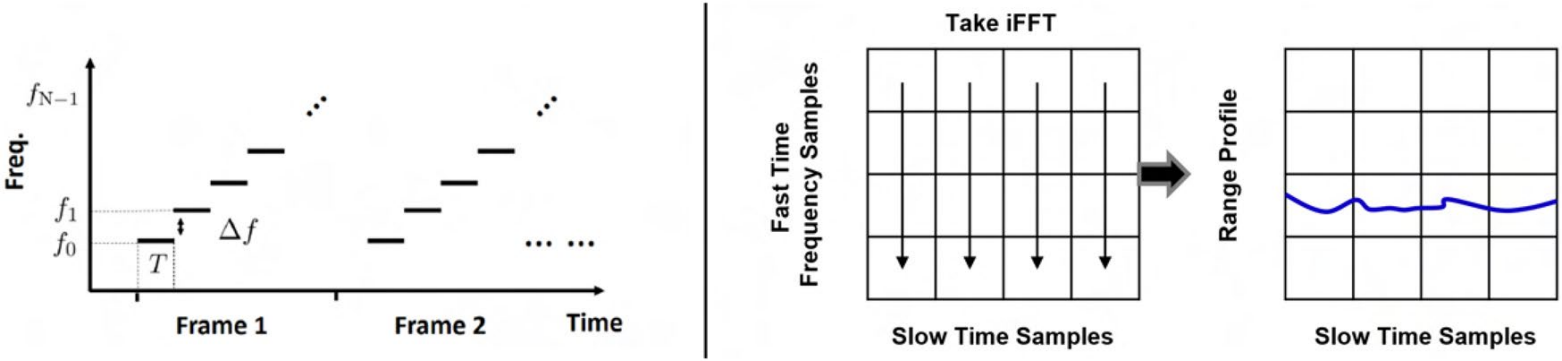
SFCW radar transmission scheme and receiver processing.

The system uses a stepped-frequency continuous wave (SFCW) radar which is an alternative architecture of the UWB radar system and operates in the frequency domain rather than time domain^53^. The SFCW radar transmits a series of discrete narrow band pulses in a stepwise to achieve a larger effective bandwidth. As such, the modulated waveform consists of a group of *N* coherent pulses with pulse duration *T*, whose frequencies are $f_\mathrm{n} = f_{0} + n \Delta f$. Assume that each SFCW waveform has *N* pulses called one SFCW frame and the center frequency of the first pulse is $f_{0}$, as illustrated in Fig. 10.

One transmitted SFCW frame is represented as a sum of *N* windowed narrow band signals,

13 $$\begin{aligned} x_\mathrm{tx}(\tau ) = \frac{1}{\sqrt{T}} \, \sum _{n} \, \mathrm{rect} \, \left( \frac{\tau - n T}{T} \right) \,\, \mathrm{e}^{{j}2\pi (f_{0} + n \Delta f)\tau }. \end{aligned}$$

The backscattered SFCW frame in baseband is modeled by concatenating the down converted received pulses. The received pulse is an attenuated and delayed version of the transmitted pulse at a nominal distance $R_{0}$. However, a slowly time-varying delay is expected due to target motion including body motion related to breathing and surface skin motion related artery pulsation, $R_{T}(t)$ is a function of slow-time *t*,

14 $$\begin{aligned} \tau _\mathrm{D}(t) = 2 \, \frac{R_{0} + R_{T}(t)}{c}, \end{aligned}$$

where *c* denotes the speed of light. The fluctuation of vital sign motion caused by breathing and heart beating is modeled as double-harmonics signal. More importantly, another time-varying component exists in $\Gamma (t, f_\mathrm{n})$ the complex reflection coefficient. In most case $T \gg \tau _{D}(t)$, then the baseband signal can be obtained by down converting and low pass filtering,

15 $$\begin{aligned} x_\mathrm{rx}(\tau ) = \frac{1}{\sqrt{T}} \, \sum _{n} \, \mathrm{rect} \, \left( \frac{\tau - n T}{T} \right) \,\, \Gamma (t, f_\mathrm{n}) \, \mathrm{e}^{{j}2\pi (f_{0} + n \Delta f)\tau _{D}(t)}. \end{aligned}$$

**Figure 11:**
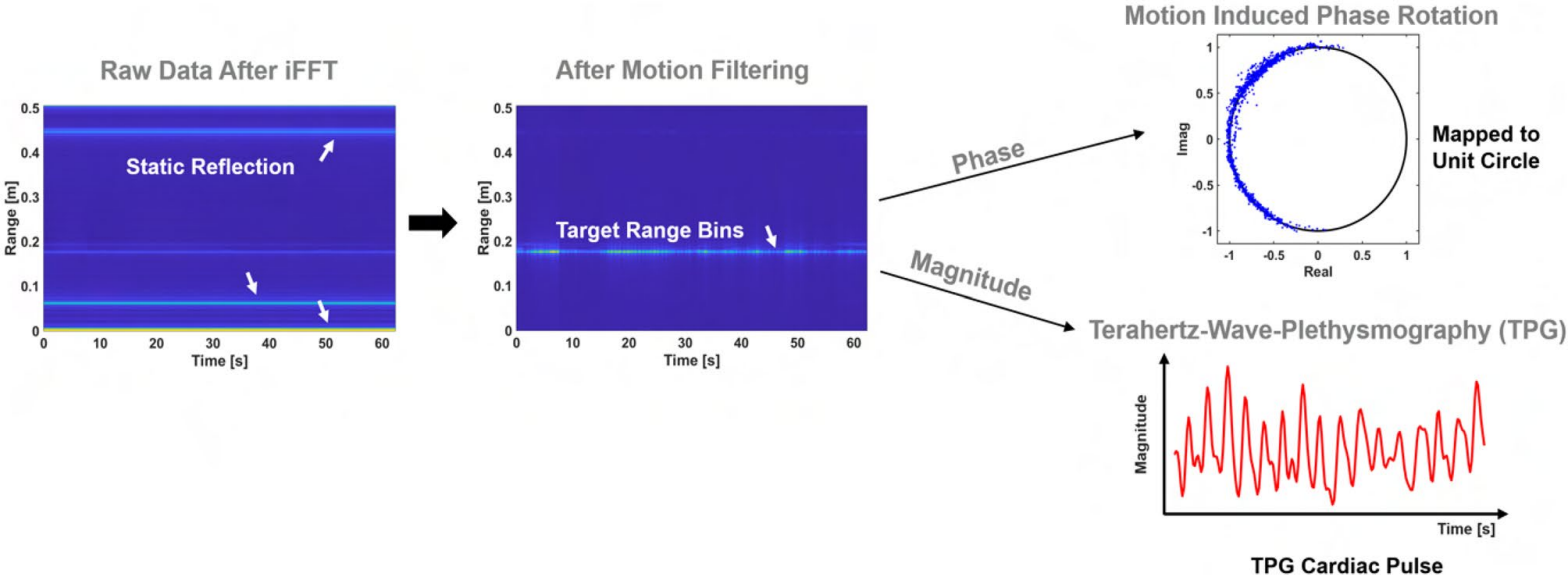
Reflectance and motion Doppler extraction in received THz data. From left to the right, they are 2-D range slow-time heatmap showing stronger static reflections, motion filtered 2-D heatmap showing only the target response, and the motion measurement extracted from the phase response caused by breathing motion displayed in the real and imaginary space and lastly the TPG measurement from the magnitude response.

The range profile is obtained by performing inverse Fourier transform of the fast time ($\tau$) frequency samples in Fig. 10. The exemplary 2-D range slow-time data matrix is displayed in Fig. 11. The processing flow involves a motion filtering to remove stronger clutterers and reveal the range bin of interest. Then, one range bin is selected for simplicity and fixed across the slow-time for further processing to extract pulse signal. For TPG analysis, the magnitude is extracted by taking the absolute operation of the range located temporal signal while the phasor is extracted by taking angular operation of the same signal. The TPG performance can be further improved and optimized by exploiting multiple range bins in the range profile offered by this wideband THz system. The robustness of the TPG method needs further investigation in the presence of motion artifacts such involuntary head motion and this problem will be explored in the future.

**Figure 12:**
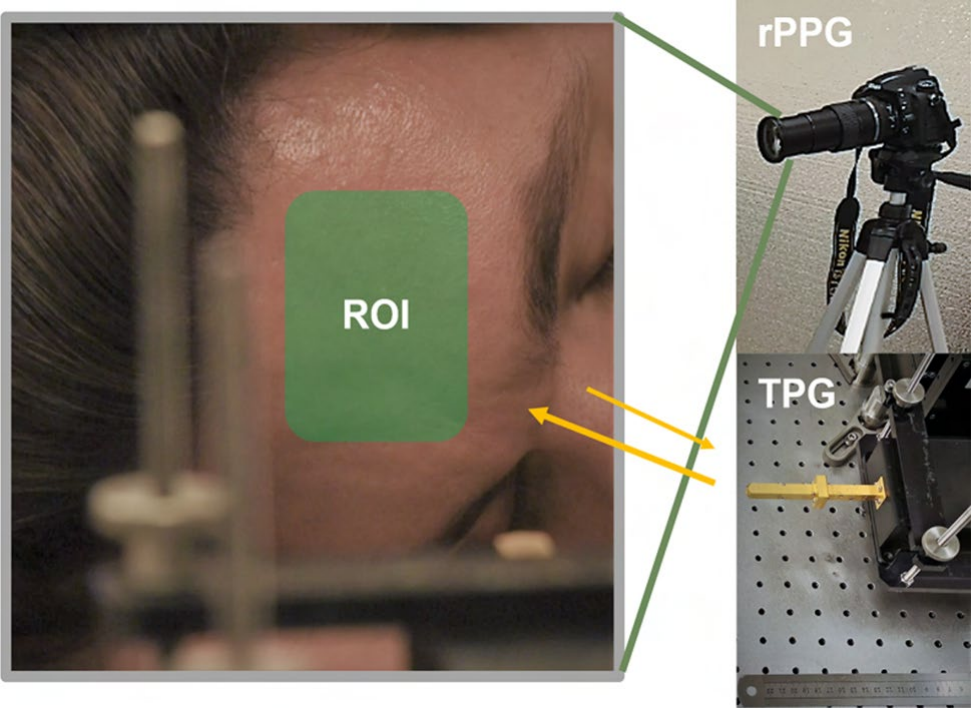
A snapshot from the recorded videos, focusing on the forehead of the test subject. The green colored area in the left figure is the manually selected area to extract rPPG signals from the sequence of image frames. rPPG measurement and TPG measurement setups are displayed in the right figure. The camera is placed about 2 m away from the subject but zoomed in to focus at the human forehead area. The THz transceiver is placed about 30 cm away from the subject. (Informed consent is obtained from this test subject for publication of identifying image.).

### Pulse sensitivity study in TPG and rPPG measurements

**Figure 13:**
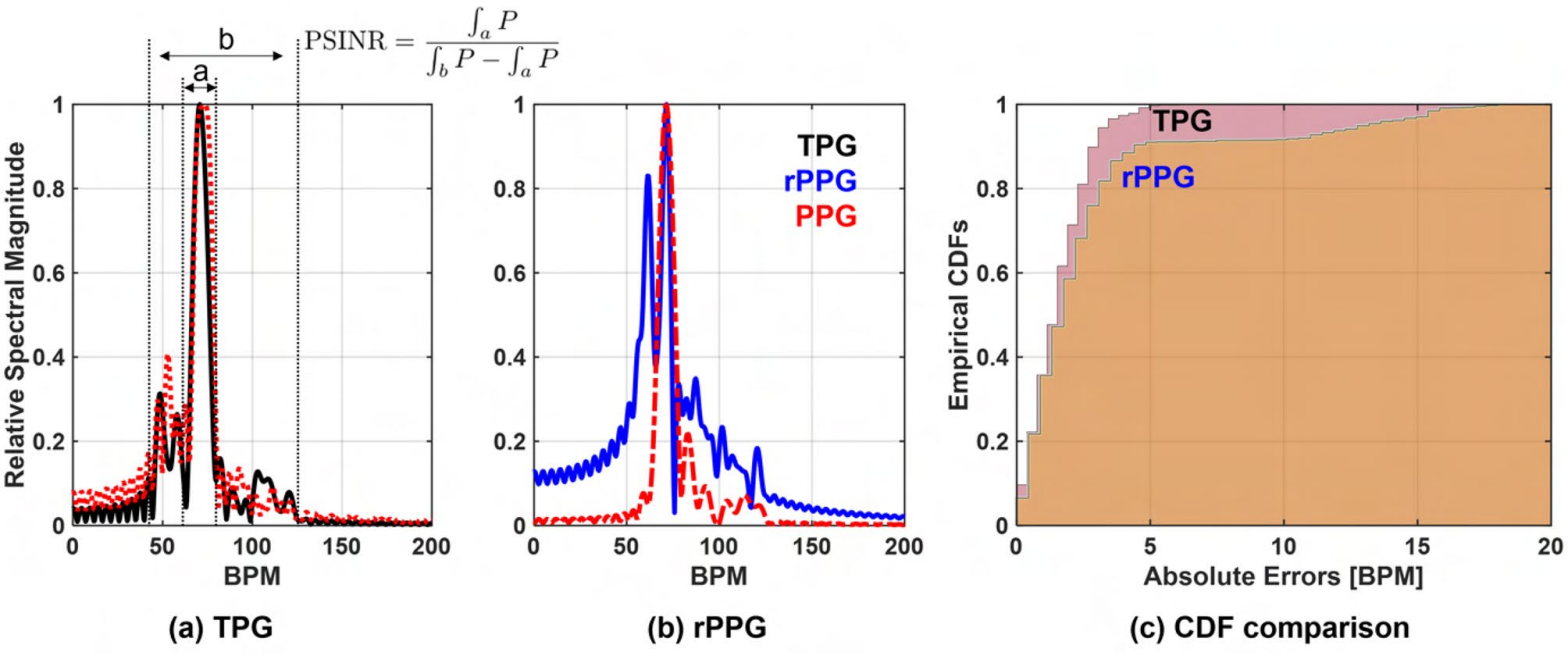
Pulse sensitivity study in the TPG and the rPPG measurements. (**a**,**b**) Visualization of pulse sensitivity in the TPG and the rPPG, and quantification of pulse sensitivity using the PSINR metric; (**c**) comparison of HR estimation performance in the form of empirical CDF from the TPG and the rPPG.

Forehead BOI is considered for pulse sensitivity analysis. The experimental setup is shown in Fig. 12. TPG and rPPG measurements are taken simultaneously from the forehead of the test subject. A snapshot of the heartbeat spectral result is shown in Fig. 13a TPG and Fig. 13b rPPG. Red curve indicates the reference PPG spectrum. The pulse measurement quality is greater in this TPG example. Pulse-signal-to-interference-plus-noise-ratio (PSINR) is used to quantitatively evaluate the pulse sensitivity. The PSINR is calculated as the ratio of the area under the power spectrum curve in a region surrounding the maximum peak in the frequency domain, to the area under the curve in the HR frequency range of interest, as illustrated in Fig. 13a. The calculated PSINR of the TPG measurement is − 1.34 dB and − 5.48 dB for the rPPG measurement. The lower PSINR value (or poorer pulse sensitivity) leads to less accurate HR estimates over time. Further more, empirical CDF of HR estimation error is used to analyze the relationship between estimation performance and pulse sensitivity. Figure 13c is obtained from a 180-s dataset. The HR is estimated from the spectral peak location. The area under the empirical CDF curve for the TPG measurement is larger than that of the rPPG measurement, and therefore better estimation performance.

In this study, the TPG outperforms the rPPG performance in terms of PSINR and CDF. The poorer HR estimation performance in rPPG can be explained by unfavored lighting condition (no dedicated lighting is applied for rPPG measurement) and further measurement distance between the camera and the test subject (about 2 m). Though it is not strictly a fair comparison between TPG and rPPG for HR estimation performance, it again highlights the usage of THz wave as non-contact reflectance method for probing cardiac pulse, which was only proven in optical waves using rPPG principle. The reported TPG and rPPG HR estimation performance can be improved further with respect to the experiment setup and algorithmic development. However, these efforts are out of scope of this paper and will be investigated in future endeavors.

### TPG penetration measurements

**Figure 14:**
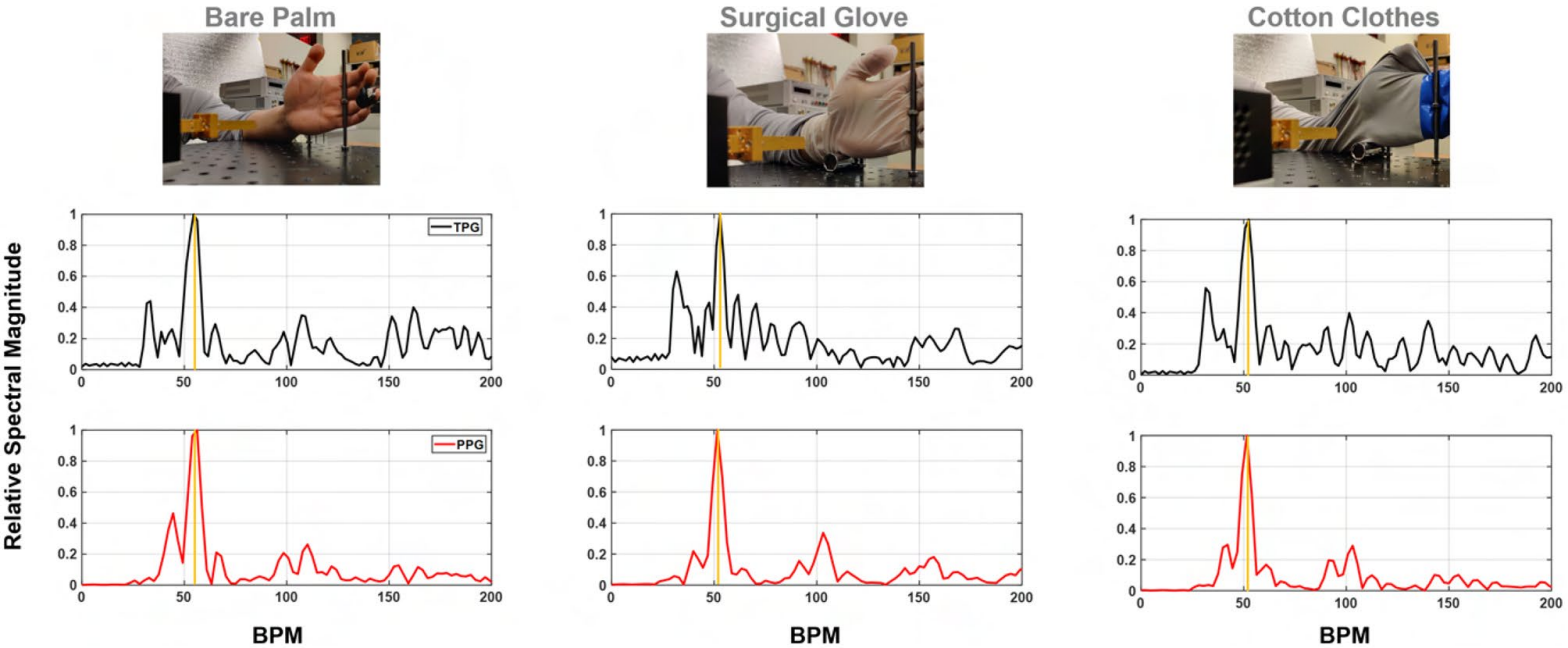
TPG penetration demonstration by comparing TPG spectra against the PPG reference spectra.

Palm BOI is considered for penetration test when skin is covered by latex glove, made of natural rubber, and long sleeve cotton clothes. The experimental setup is shown in Fig. 14 top row. These experiments are conducted to validate the penetration capability of the proposed TPG method at palm. In Fig. 14, the first row displays a snapshot of the experiment scene for a bare palm, a covered palm with a surgical glove, and a covered palm with cotton clothes. For both cases, the TPG measurement can estimate heart rate accurately. The black curve denotes TPG while the red curve is the corresponding PPG reference. 20 s of data is processed to generate the following results. The bare palm example is also provided as a baseline for the two penetration examples. For all cases, the major spectral energy is centered around the heartbeat as indicated by the reference PPG signal. Additionally, the quantitative measure of TPG penetrability is calculated in the forms of the PSINR metric. The detected spectral pulse strength is computed and summarized in Table 4. Compared to bare palm measurement, the TPG results via glove and clothes in the study experience $2.81 - (-0.91) = 3.71$ (dB) and $2.81 - (-4.65) = 7.46$ (dB) power loss due to material penetration.

**Table 4:** TPG penetration comparison.

	Palm	Glove	Clothes
PSINR (dB)	2.81	$-$ 0.91	$-$ 4.65

### Subjects and experimental protocol

Measurements were taken from 4 human test subjects. Totally our database contains 8540 s of data, comprising asynchronized raw data of PPG, rPPG and data derived from radar. All measurements were recorded under standardized conditions, seated comfortably in an arm chair with back support and breathing normally at leisure. During the experiments, the distance between antenna and BOIs varied from 10 to 60 cm. Additionally, predefined interventions were considered, changing measurement positions including sitting, standing and lying down, changing heartbeat variability by physical exercising for 5 min before measurements, changing breathing pattern by holding breathing for 15–30 s. Data acquisition was acquired following the study protocol over different BOIs, such as finger, forehead, inner elbow, which are illustrated in Fig. 8.

### Ethics approval

The study was approved by the ethics committee of the Arizona State University. All research was performed in accordance with relevant guidelines and regulations. The informed consent was obtained from all subjects in human trials.

## Data Availability

The data that support the findings of this study are available from the corresponding authors upon reasonable request.
